# Ultrasound-Guided Percutaneous Electrolysis and Eccentric Exercises for Subacromial Pain Syndrome: A Randomized Clinical Trial

**DOI:** 10.1155/2015/315219

**Published:** 2015-11-15

**Authors:** José L. Arias-Buría, Sebastián Truyols-Domínguez, Raquel Valero-Alcaide, Jaime Salom-Moreno, María A. Atín-Arratibel, César Fernández-de-las-Peñas

**Affiliations:** ^1^Department of Physical Therapy, Universidad Francisco de Vitoria, Carretera Pozuelo a Majadahonda, Km 1.800, Pozuelo de Alarcón, 28223 Madrid, Spain; ^2^Cátedra de Investigación y Docencia en Fisioterapia, Terapia Manual y Punción Seca, Universidad Rey Juan Carlos, Avenida de Atenas s/n, Alcorcón, 28922 Madrid, Spain; ^3^Department of Physical Therapy, Hospital Universitario Gregorio Marañón, Doctor Esquerdo 46, 28007 Madrid, Spain; ^4^Department of Physical Therapy, Universidad Camilo Jose Cela, Urb. Villafranca del Castillo, Calle Castillo de Alarcón 49, Villanueva de la Cañada, 28692 Madrid, Spain; ^5^Facultad de Enfermería, Fisioterapia y Podología, Universidad Complutense de Madrid, Avenida Séneca 2, 28040 Madrid, Spain; ^6^Department of Physical Therapy, Occupational Therapy, Rehabilitation and Physical Medicine, Universidad Rey Juan Carlos, Avenida de Atenas s/n, Alcorcón, 28922 Madrid, Spain; ^7^Grupo Excelencia Investigadora URJC-Banco Santander Referencia N°30VCPIGI03, Investigación Traslacional en el Proceso de Salud-Enfermedad (ITPSE), Universidad Rey Juan Carlos, Avenida de Atenas s/n, Alcorcón, 28922 Madrid, Spain

## Abstract

*Objective*. To compare effects of ultrasound- (US-) guided percutaneous electrolysis combined with an eccentric exercise program of the rotator cuff muscles in subacromial pain syndrome.* Methods*. Thirty-six patients were randomized and assigned into US-guided percutaneous electrolysis (*n* = 17) group or exercise (*n* = 19) group. Patients were asked to perform an eccentric exercise program of the rotator cuff muscles twice every day for 4 weeks. Participants assigned to US-guided percutaneous electrolysis group also received the application of galvanic current through acupuncture needle on each session once a week (total 4 sessions). Shoulder pain (NPRS) and disability (DASH) were assessed at baseline, after 2 sessions, and 1 week after the last session.* Results*. The ANOVA revealed significant Group*∗*Time interactions for shoulder pain and disability (all, *P* < 0.01): individuals receiving US-guided percutaneous electrolysis combined with the eccentric exercises experienced greater improvement than those receiving eccentric exercise alone.* Conclusions*. US-guided percutaneous electrolysis combined with eccentric exercises resulted in small better outcomes at short term compared to when only eccentric exercises were applied in subacromial pain syndrome. The effect was statistically and clinically significant for shoulder pain but below minimal clinical difference for function. Future studies should investigate the long-term effects and potential placebo effect of this intervention.

## 1. Introduction

Shoulder pain is highly prevalent and a common reason for individuals to seek physical therapy. The one-year prevalence of shoulder pain ranges from 20% to 50% in the general population [1, 2]. Among the different causes explaining shoulder pain, the most prevalent diagnosis is rotator cuff pathology and subacromial pain syndrome [3, 4]. Walker-Bone et al. found a prevalence of 4.5% in men and 6.1% in women of rotator cuff pathology [5].

Conservative management is the initial treatment option for individuals suffering from shoulder pain; however, the most appropriate treatment strategies are still unclear. The Dutch clinical guideline discusses the potential use of some physical therapy therapeutic approaches such as education, manual therapy, exercises, electrotherapy modalities (i.e., ultrasound and laser) and taping interventions, and medical management consisting in nonsteroidal anti-inflammatory medication (NSAID) or injections [6]. This guideline advises regarding the use of treatment strategies with high levels of evidence (level 1 or 2). In shoulder pain, only steroids and exercises reached this level [6]. In fact, therapeutic exercise is probably the conservative strategy exhibiting the highest level of evidence for the management of shoulder pain conditions [7]. The Cochrane review found that exercise was effective at short term in rotator cuff disease/subacromial pain syndrome [8]; however, more recent reviews have concluded that although the role of therapeutic exercise in the treatment of shoulder pain is promising, caution should be considered due to the paucity of high quality research and limitations in the outcomes [9, 10].

**Figure 1:**
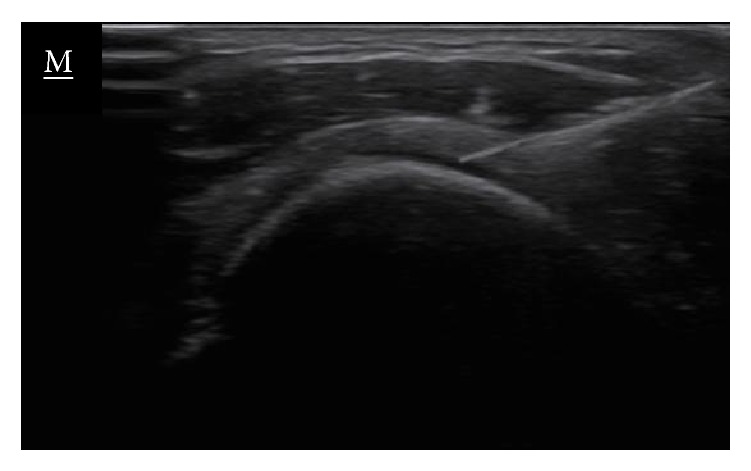
Long-axis grey-scale ultrasound image displaying the supraspinatus tendon during needle placement showing the echogenic needle during the application of US-guided percutaneous electrolysis.

In the last years, the application of a galvanic current through an acupuncture needle has been advocated for the management of tendinopathies [11]. This technique consists of the combination of mechanical (needle) stimulation and electrical (galvanic current) stimulation as a method to provide a controlled microtrauma to the affected structure, in this case the tendon (Figure 1). Different commercial names are proposed: intratissue percutaneous electrolysis [EPI^®^], therapeutic percutaneous electrolysis [EPTE^®^], or Physio Invasiva^®^ being the common term of percutaneous electrolysis. The needle is directed into the tendon under visualisation using an ultrasound (US); therefore, the intervention is US-guided.

The theoretical framework of the percutaneous electrolysis technique is to induce an inflammatory response by inducing a nonthermal electrolytic reaction in the targeted tissue (tendon) through a cathodic flow [12]. This controlled local inflammatory response will facilitate an organic reaction leading to the regeneration of the injured tendon [12]. Some preliminary studies demonstrated that the application of percutaneous electrolysis may be effective in patellar and elbow tendinopathies [13, 14]. Nevertheless, these studies did not include a control group.

To the best of the authors' knowledge, no randomized clinical trial investigating the effects of the percutaneous electrolysis technique has been conducted in individuals with shoulder pain conditions. Therefore, the purpose of this randomized clinical trial was to compare the effects of combined US-guided percutaneous electrolysis with an exercise program of the rotator cuff musculature to an exercise program of the rotator cuff muscles alone on pain and disability in subjects with subacromial pain syndrome. We hypothesized that subjects receiving US-guided percutaneous electrolysis combined with an eccentric exercise program of the rotator cuff musculature will exhibit higher improvements in pain and disability than those receiving the eccentric exercise program alone.

## 2. Methods

### 2.1. Participants

A randomized single blind clinical trial was conducted (this trial is registered with NCT02196948). Consecutive subjects with a diagnosis of subacromial pain syndrome presenting to a physical therapy clinic in Madrid (Spain) from July 2014 to November 2014 were screened for inclusion in this study. To be included in the study, individuals had to fulfil all the following criteria: (1) unilateral shoulder complaints with duration of at least 3 months, (2) an intensity of at least 4 on 11-point numerical pain rating scale (NPRS) during arm elevation, (3) a positive painful arc test during abduction [15], (4) at least one positive test of Hawkins-Kennedy test, Neer's sign, empty can test, drop arm, and lift-off test [16], and (5) positive findings of rotator cuff/supraspinatus tendinopathy on magnetic resonance imaging (MRI) [17].

Patients were excluded if they exhibited any of the following criteria: (1) bilateral shoulder symptoms, (2) age below 18 years or over 65 years, (3) history of shoulder fractures or dislocation, (4) cervical radiculopathy, (5) previous interventions with steroid injections, (6) fibromyalgia syndrome, (7) previous history of shoulder or neck surgery, or (8) any type of intervention for the neck-shoulder area in the previous year. The study was approved by the local Ethics Committee of Universidad Rey Juan Carlos, Spain (URJC 22/2014) and it was conducted following the Helsinki Declaration. All participants signed an informed consent prior to their inclusion.

### 2.2. Outcome Measures

The primary outcome measure of this trial was the intensity of shoulder pain. A 10-point numerical pain rating scale (NPRS; 0: no pain, 10: maximum pain) was used to assess the pain status: (a) current level of shoulder pain, (b) worst level of shoulder pain experienced in the preceding week, and (c) lowest level of pain experienced in the preceding week [18]. Mintken et al. found that the minimal clinically important difference (MCID) for the NPRS in patients with shoulder pain was 1.1 points [19].

The secondary outcome included disability and it was assessed with the Disabilities of the Arm, Shoulder, and Hand (DASH) questionnaire [20]. It consists of a region-specific questionnaire assessing disability and symptoms in individuals suffering from musculoskeletal pain disorders of the upper extremity. It includes 30 items assessing (1) degree of difficulty during the preceding week in performing several physical activities because of problems in a upper extremity (21 items), (2) severity of each of the symptoms of pain, activity-related pain, tingling, weakness, and stiffness (5 items), and (3) the problem's effect on social activities, work, and sleep and its psychological impact (4 items). Each item is answered on a 5-point scale ranging from 1 (no difficulty to perform, no symptom, or no impact) to 5 (unable to do, very severe symptom, or high impact). The responses to the 30 items are summed to form a raw score that is then converted to a scale from 0 to 100 with the formula, [(sum of score/*n*) − 1] × 25, where *n* is the number of completed responses. A higher score reflects greater disability [20]. It has been recently found that the MCID for the DASH was 10.81 points [21].

### 2.3. Randomization

Following the baseline examination, patients were randomly assigned to receive either US-guided percutaneous electrolysis combined with eccentric exercise program of the rotator cuff musculature (electrolysis group) or eccentric exercise program of the rotator cuff muscles alone (exercise group).

Concealed allocation was performed using a computer-generated randomized table of numbers created prior to data collection with EPIDAT^®^ 3.1. Individual and sequentially numbered index cards with random assignment were prepared. The index cards were folded and placed in sealed opaque envelopes. A second external researcher opened the envelope and proceeded with treatment according to the group assignment.

Each group was treated by a clinician with more than 10 years of experience in the management of shoulder pain problems. There is no consensus on the frequency of exercises for individuals with shoulder complains with a frequency ranging from twice weekly to daily [22]. Furthermore, the review concluded that supervised exercise therapy should progress toward home exercises [22]. Therefore, all participants of this study attended a physical therapy clinic once per week for 4 weeks for supervised training sessions. On each session, the eccentric exercise program was explained (first session) and properly revised by the clinician. During the period, patients were asked to perform the exercise program on an individual basis twice every day for 4 weeks. Subjects assigned to the electrolysis group received the application of US-guided percutaneous electrolysis on each session once a week (4 sessions in total).

Outcomes were assessed at baseline, after 2 sessions (middle follow-up), and 1 week after the last session (posttreatment follow-up).

### 2.4. Eccentric Exercise Program

**Figure 2:**
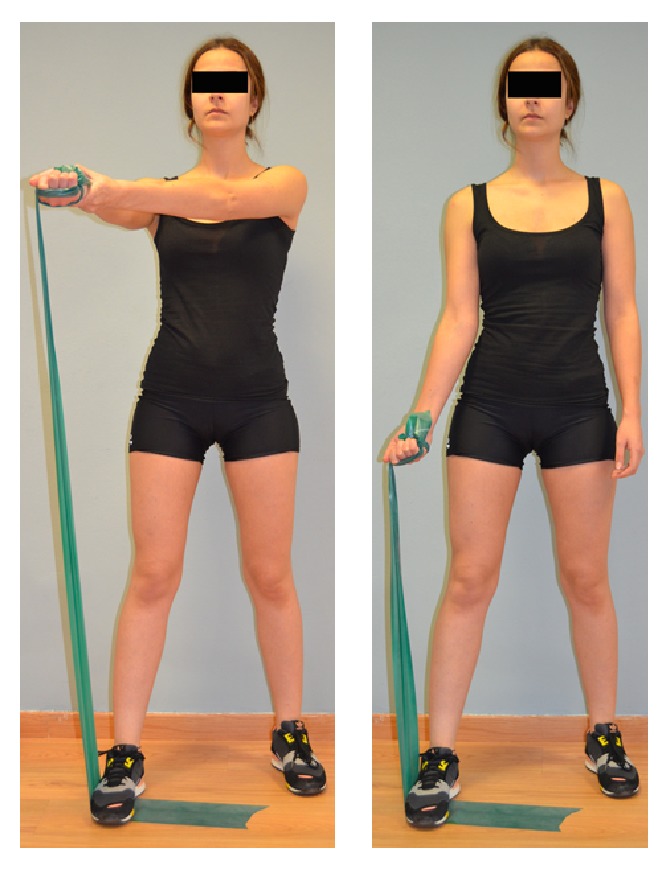
Eccentric exercise of the supraspinatus muscle. Patients were asked to do a normal abduction (concentric phase) and a slow return to the initial position (eccentric phase).

**Figure 3:**
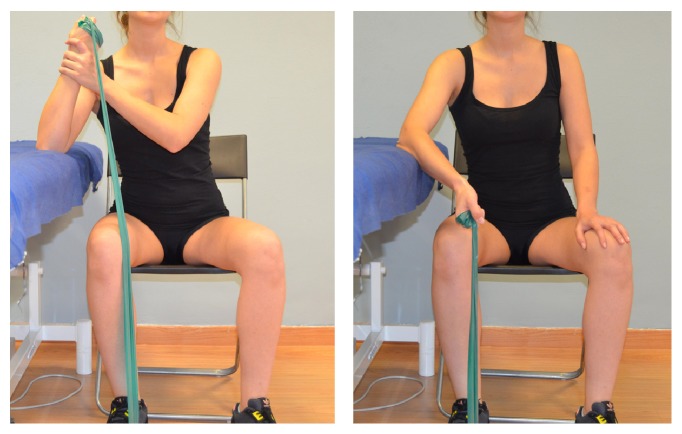
Eccentric exercise of the infraspinatus muscle. Patients were asked to do a normal external rotation (concentric phase) and a slow return to the initial position (eccentric phase).

**Figure 4:**
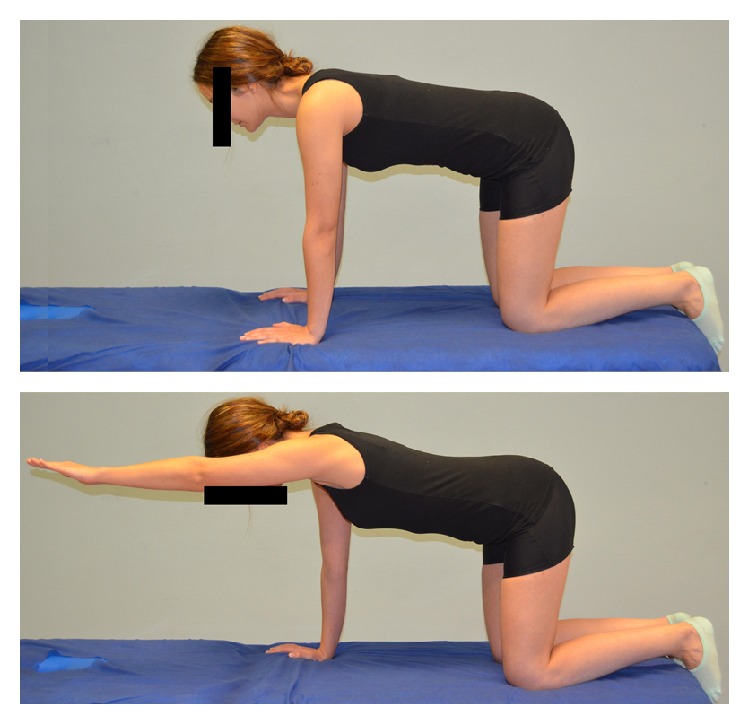
Stabilization exercise of the scapular musculature in kneeling position.

There is no consensus on the eccentric exercises to be applied on individuals with subacromial pain syndrome, although some authors have recommended that they should be specific and should be of low intensity and high frequency [23]. Eccentric exercises were performed in 3 sets of 10 repetitions. Each repetition included first the concentric phase, and the eccentric phase was slowly conducted. The eccentric program consisted of 3 exercises, focusing on the supraspinatus (Figure 2), infraspinatus (Figure 3), and scapular (Figure 4) muscles. The exercise program was taught by a physiotherapist in the first session and monitored in the subsequent sessions. Participants were asked to perform the exercise program on an individual basis twice every day for 4 weeks

### 2.5. US-Guided Percutaneous Electrolysis

Individuals assigned to this group received, in addition to the eccentric exercise program, one session of US-guided percutaneous electrolysis per week over 4 weeks. The technique was applied using a specifically developed medically certified device (EPTE^®^ V01, classification IIa, Ionclinics, Valencia, Spain), which produces modulated galvanic electricity through the negative electrode cathodic flow. The galvanic current is applied using acupuncture needles of different lengths (0.3 mm in diameter). The intensity of the intervention is adjusted by modifying the duration of application or microamperes (*μ*A) of the device. This technique is applied to the patient without pain. The technique was applied under US guidance (US system hand-carried colour Doppler Mindray^®^ M7) on the clinically relevant area, that is, supraspinatus tendon, using an intensity of 350 *μ*A during 1.2 min.

**Figure 5:**
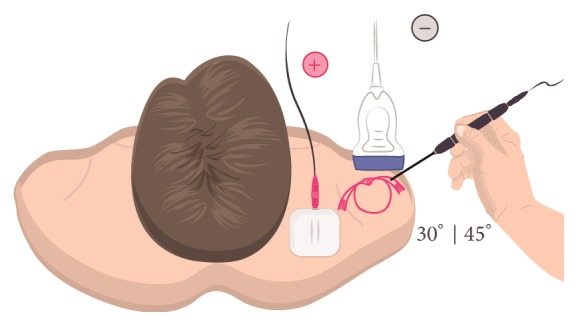
Needle approach during the application of US-guided percutaneous electrolysis. The figure shows the location of cathode (targeting the supraspinatus tendon) and anode (over the upper trapezius muscle) electrodes for application of percutaneous electrolysis. The transducer is placed on the supraspinatus tendon and the needle is inserted in the centre of the transducer in a long-axis position at an angle of about 30–45° to the skin surface, depending on the target area, and then advanced parallel to the sound beam.

**Figure 6:**
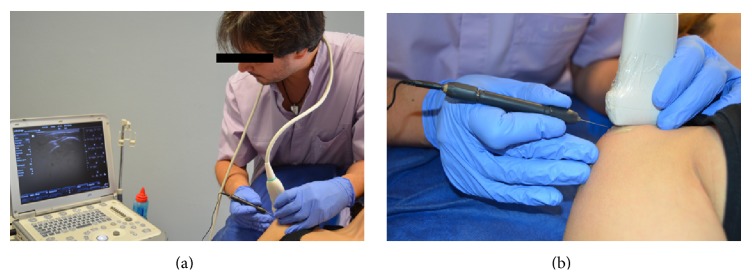
Application of US-guided percutaneous electrolysis on a real patient with the clinician following the application on the ultrasound screen (a) and a detail of the application (b).

The patient was placed in a supine position with the affected shoulder placed in internal rotation. The anterior part of the shoulder was sterilised with isopropyl alcohol and the sonographic transducer, enclosed in a sterile cover over sterile (Tegaderm Film, 3M, 10 cm × 12 cm) applied gel, was placed at the anatomical projection of the supraspinatus tendon (Figure 5). A 0.3*∗*25 mm acupuncture needle (Agupunt, Barcelona, Spain) was inserted at an 80° angle to the skin with the needle tip directed towards the supraspinatus tendon (Figure 6).

### 2.6. Sample Size Determination

The sample size was calculated using Ene 3.0 software (Autonomic University of Barcelona, Spain). The calculations were based on detecting differences of 1.1 units in the primary outcome (NPRS) at postdata (MCID), assuming a standard deviation of 1.05 [19], a 2-tailed test, an alpha level (*α*) of 0.05, and a desired power (*β*) of 80%. The estimated desired sample size was calculated to be at least 16 subjects per group.

### 2.7. Adverse Events

All participants were asked to report any adverse events that they experienced during the study. An adverse event was defined as sequelae of medium term in duration with any symptom perceived as distressing and unacceptable and required further treatment [24].

### 2.8. Statistical Analysis

Statistical analysis was performed using SPSS statistical software, version 18.0, and was conducted according to the intention to treat analysis principle. Mean, standard deviations, and/or 95% confidence intervals were calculated for each variable. Kolmogorov-Smirnov test showed a normal distribution of the data (*P* > 0.05). Baseline demographic and clinical variables were compared between groups using independent Student's *t*-tests for continuous data and *χ* ^2^ tests of independence for categorical data. A 3 × 2 repeated measures ANOVA with time (baseline, middle follow-up, and posttreatment) as the within-subjects factor and group (electrolysis or exercise) as the between-subjects factor was used to determine the effects of the intervention on NPRS and DASH. The hypothesis of interest was the Group*∗*Time interaction with Bonferroni-corrected alpha of 0.015 (3 moments). To enable comparison of effect sizes, standardized mean score differences (SMDs) were calculated by dividing mean score differences between electrolysis group and comparison group (exercise group) by the pooled standard deviation.

## 3. Results

**Figure 7:**
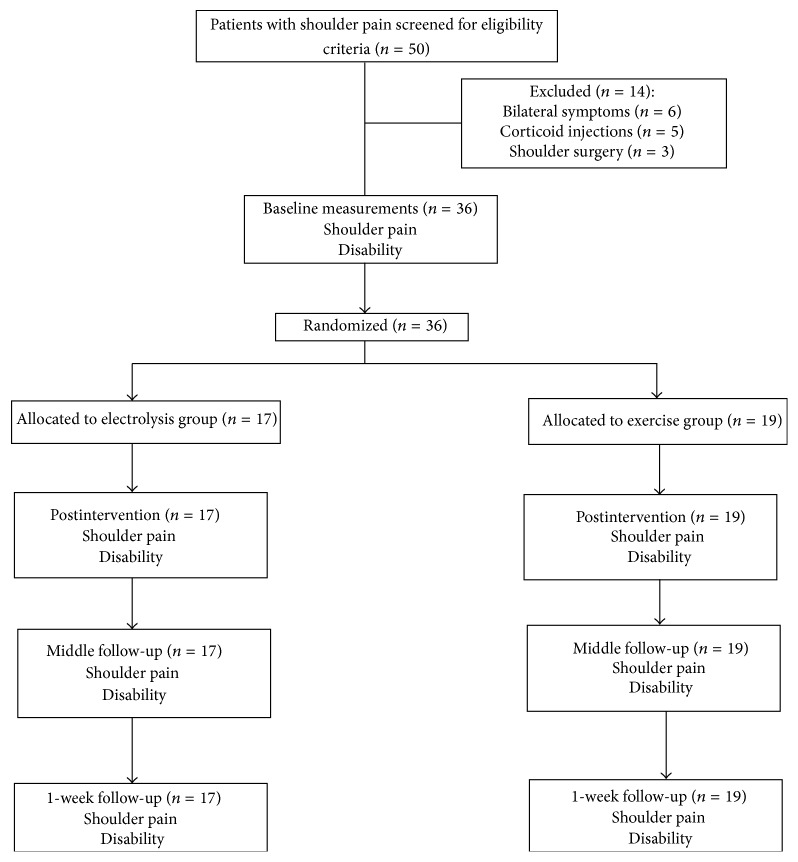
Flow diagram of patients throughout the course of the study.

Fifty consecutive patients with shoulder pain were screened for eligibility criteria. Thirty-six patients (mean ± SD age: 58 ± 7 years; 75% female) satisfied the eligibility criteria, agreed to participate, and were randomized into electrolysis (*n* = 17) group or exercise (*n* = 19) group. The reasons for ineligibility can be found in Figure 7, which provides a flow diagram of patient recruitment and retention. Baseline features between groups were similar for all variables (Table 1).

The 3 × 2 mixed model ANOVA revealed significant Group*∗*Time interactions for the current level of shoulder pain (*F* = 10.447; *P* = 0.003) and the worst level of shoulder pain experienced in the preceding week (*F* = 12.269; *P* = 0.001) but not for the lowest level of pain experienced (*F* = 0.204; *P* = 0.655): those individuals receiving US-guided percutaneous electrolysis and eccentric exercise program experienced greater decrease in pain than those receiving eccentric exercises alone (Table 2). There was a main effect for time with both groups showing similar decreases in the lowest level of pain (*F* = 49.874; *P* < 0.001). Between-groups effect sizes were large for the current level of pain at both follow-up periods (SMD > 2.01) and at posttreatment follow-up for the worst level of pain (SMD: 3.20) in favor of the US-guided percutaneous electrolysis group. Table 2 provides baseline, middle follow-up, and posttreatment follow-up as well as within-groups differences with their associated 95% CI for pain outcomes.

The 3 × 2 ANOVA also revealed a significant Group*∗*Time interaction for DASH (*F* = 7.882; *P* = 0.008): patients receiving percutaneous electrolysis and eccentric exercises showed a greater decrease in disability than those receiving eccentric exercise program alone (Table 2). Between-groups effect sizes were large at both follow-up periods (SMD > 2.52) in favor of the US-guided percutaneous electrolysis group. Table 2 provides baseline, middle follow-up, and posttreatment follow-up as well as within-groups differences with their associated 95% CI for disability.

In our study, 6 patients assigned to the US-guided percutaneous electrolysis group (35%) experienced local soreness at the supraspinatus tendon after the first 2 treatments. Posttreatment soreness resolved spontaneously within 24–36 hours without any intervention. In addition, 30 patients (83%) experienced delayed muscle soreness (DOMS) in the shoulder muscles during the 1st week of the eccentric exercise program, but it disappeared during the second week spontaneously.

## 4. Discussion

The results of the current randomized clinical trial suggest that the combination of US-guided percutaneous electrolysis and an eccentric exercise program resulted in better outcomes at short term compared to when only exercises were applied in individuals with subacromial pain syndrome. We could anticipate that the benefit of adding US-guided percutaneous electrolysis for the management of subacromial pain syndrome may be clinically relevant as noted by significant between-group effect sizes, particularly in the current level and the worst level of shoulder pain. In addition, although significant between-group differences were also found in disability, changes were lower than MCID at posttreatment. In fact, while both groups exhibited significant improvements from baseline, we cannot be certain if these changes were result of the interventions applied or were simply due to the passage of time since we did not include a control group that received no intervention.

The Dutch clinical guideline supports the use of exercises for the management of patients with subacromial pain syndrome [6]. In addition, several systematic reviews have also supported the effectiveness of exercises in this pain condition [7, 8]. Our study also supports the effectiveness of eccentric exercises for the management of pain and disability in individuals with subacromial pain syndrome since both groups exhibited clinical improvements in shoulder pain and disability. Within-group change scores and their 95% confidence interval bounds in both groups surpassed the MCID for pain [19] and disability [20], supporting a clinical effect of the eccentric exercise program at a short-term follow-up period.

The novelty of this clinical trial was the application of US-guided percutaneous electrolysis for the management of subacromial pain syndrome. There is preliminary evidence suggesting that the application of percutaneous electrolysis may be effective for some tendinopathies [13, 14]; however, no randomized clinical trial investigating this therapeutic approach has been yet conducted. The current study is the first randomized clinical trial investigating the effectiveness of percutaneous electrolysis. Our trial found that subjects receiving US-guided percutaneous electrolysis in addition to an eccentric exercise program exhibited better outcomes in pain and disability than those individuals who did not receive the US-guided percutaneous electrolysis intervention. In this case, between-group change scores surpassed the MCID for current and worst levels of pain in favor of the US-guided percutaneous electrolysis group. Nevertheless, lower bound of 95% confidence intervals falls over MCID in some pain outcomes at some follow-up periods. Similarly, between-group scores on disability, although statistically significant, did not surpass the MCDI. Therefore, clinical relevance of the current results should be considered with caution.

The exact therapeutic mechanism by which US-guided percutaneous electrolysis exerts its effects remains to be elucidated, and both mechanical and biochemical effects are currently proposed. It has been hypothesized that percutaneous electrolysis provokes tenocyte disruption as well as local inflammatory processes occurring in the tendon which enable phagocytosis and repair of the affected tissue [7, 8]. This hypothesis was partly supported by an animal study demonstrating that the application of percutaneous electrolysis produced an increase in anti-inflammatory and angiogenic molecular mechanisms in collagenase-induced tendon injury of a rat since significant increases in the expression of cytochrome C, vascular endothelial growth factor, and peroxisome proliferator-activated receptor gamma were seen [12].

Since tendon abnormalities of the rotator cuff include degeneration and disordered arrangement of collagen fibers, inflammatory cell infiltration, tenocytes disruption, and vascularity changes, some authors have proposed considering biological options such as percutaneous electrolysis in addition to biomechanical (eccentric exercises) treatment for rotator cuff pathology [25]. It is therefore possible that application of percutaneous electrolysis previously to eccentric exercises can potentially affect the mechanical properties of the tendon permitting better dynamics of the tissue.

There are a number of limitations of the current study that should be considered. First, only 2 clinicians performed the US-guided percutaneous electrolysis intervention which might limit the generalizability of the current results. Second, we only assessed outcomes at a short-term follow-up of 1 week and cannot be certain if these differences remained in the long term. This is critical to determining the potential clinical effect of this technique. Third, we did not include a control group, so it cannot be determined if improvements observed in both groups can be attributed to the interventions or simply the passage of time, although this is unlikely since the included patients had chronic symptoms. Similarly, for both groups, the influence of placebo effect is unknown as we did not have a group who received a sham intervention, particularly a sham electrolysis approach [26]. It is possible that consecutive applications of percutaneous electrolysis have a placebo effect because of the use of technology such as US. Future studies including multiple therapists, a sham percutaneous electrolysis approach group, and long-term follow-ups should be conducted to determine the best multimodal therapeutic option for the treatment of shoulder pain.

## 5. Conclusion

The results of the current randomized clinical trial suggest that the application of US-guided percutaneous electrolysis combined with an eccentric exercise program resulted in small better outcomes on pain and disability at short term compared to when only exercises were applied in subjects with subacromial pain syndrome. The effect was statistically and clinically significant for pain intensity but below the MCDI for disability. Furthermore, while both groups exhibited significant improvements from baseline, we cannot be certain if these changes were result of the interventions applied or were simply due to the passage of time since we did not include a control group that received no intervention. Future studies are clearly needed to investigate the effects of this therapeutic intervention for tendinopathies.

## Figures and Tables

**Table 1:** Baseline demographics for both groups^*∗*^.

	Electrolysis group (*n* = 17)	Exercise group (*n* = 19)	*P* values
Gender (male/female)	4/13	5/14	0.847
Age (years)	58 ± 7	57 ± 6	0.629
Affected side (left/right)	8/9	9/10	0.738
Time duration (months)	11.2 ± 2.7	10.6 ± 2.6	0.483
Mean pain intensity (0–10)	7.0 ± 0.9	6.8 ± 0.7	0.581
Worst pain intensity (0–10)	8.2 ± 1.3	8.1 ± 1.4	0.883
Lowest pain intensity (0–10)	4.8 ± 1.2	5.0 ± 1.0	0.639
DASH (0–100)	57.4 ± 4.0	57.6 ± 9.2	0.934

^*∗*^Data are mean ± SD except for gender.

DASH: Disabilities of the Arm, Shoulder, and Hand.

**Table 2:** Outcome data for shoulder pain and disability.

Mean pain intensity (0–10)	Pretreatment	Middle follow-up	Posttreatment
Electrolysis group^*∗*^	7.0 ± 0.9	3.8 ± 1.0	1.4 ± 1.2
Exercise group^*∗*^	6.8 ± 0.7	5.1 ± 1.2	3.1 ± 2.1
Within-group change score from baseline^#^			
Electrolysis group^*∗∗*^		−3.2 (−3.9, −2.5)^##^	−5.6 (−6.4, −4.7)^##^
Exercise group^*∗∗*^		−1.7 (−2.2, −1.3)^##^	−3.7 (−4.6, −2.9)^##^
Between-group difference in change score^*∗∗*^		1.5 (0.7, 2.2)^##^	1.9 (0.7, 3.1)^##^

Worst pain intensity (0–10)	Pretreatment	Middle follow-up	Posttreatment

Electrolysis group^*∗*^	8.2 ± 1.3	5.1 ± 2.4	2.3 ± 1.2
Exercise group^*∗*^	8.1 ± 1.4	5.3 ± 2.5	4.5 ± 2.4
Within-group change score from baseline^#^			
Electrolysis group^*∗∗*^		−3.1 (−4.4, −1.7)^##^	−5.9 (−6.7, −5.0)^##^
Exercise group^*∗∗*^		−2.8 (−3.8, −1.6)^##^	−3.6 (−4.6, −2.5)^##^
Between-group difference in change score^*∗∗*^		0.3 (−1.4, 2.0)	2.3 (1.2, 3.3)^##^

Lowest pain intensity (0–10)	Pretreatment	Middle follow-up	Posttreatment

Electrolysis group^*∗*^	5.8 ± 1.2	3.5 ± 2.3	1.2 ± 1.1
Exercise group^*∗*^	5.0 ± 1.0	3.9 ± 2.3	1.3 ± 1.2
Within-group change score from baseline^#^			
Electrolysis group^*∗∗*^		−2.3 (−3.4, −1.8)^##^	−4.6 (−5.8, −3.5)^##^
Exercise group^*∗∗*^		−2.1 (−2.9, −1.7)^##^	−3.7 (−4.5, −2.8)^##^
Between-group difference in change score^*∗∗*^		0.2 (−1.1, 1.4)	1.1 (−0.3, 1.5)

DASH (0–100)	Pretreatment	Middle follow-up	Posttreatment

Electrolysis group^*∗*^	57.4 ± 4.0	26.1 ± 10.3	11.1 ± 8.8
Exercise group^*∗*^	57.6 ± 9.2	38.5 ± 11.4	20.8 ± 7.4
Within-group change score from baseline^#^			
Electrolysis group^*∗∗*^		−31.3 (−35.8, −26.7)^##^	−46.3 (−52.2, −40.5)^##^
Exercise group^*∗∗*^		−19.1 (−24.2, −14.0)^##^	−36.8 (−42.2, −31.4)^##^
Between-group difference in change score^*∗∗*^		12.2 (5.6, 18.9)^##^	9.5 (1.9, 17.2)^##^

^*∗*^Data are means ± standard deviations; ^*∗∗*^data are means (95% confidence intervals).

^#^Compared to pretreatment; ^##^statistically significant differences (*P* < 0.01).

DASH: Disabilities of the Arm, Shoulder, and Hand.
